# Utilization of *n*-alkane and roles of lipid transfer proteins in *Yarrowia lipolytica*

**DOI:** 10.1007/s11274-023-03541-3

**Published:** 2023-02-14

**Authors:** Ryouichi Fukuda

**Affiliations:** 1 Department of Biotechnology, The University of Tokyo, Yayoi 1-1-1, Bunkyo-ku, Tokyo, 113-8657 Japan; 2 Collaborative Research Institute for Innovative Microbiology, The University of Tokyo, Yayoi 1-1-1, Bunkyo-ku, Tokyo, 113-8657 Japan

**Keywords:** *Yarrowia lipolytica*, *n*-Alkane, Fatty acid, Dimorphism, Lipid transfer protein

## Abstract

*Yarrowia lipolytica*, a dimorphic yeast belonging to the Ascomycota, has potent abilities to utilize hydrophobic compounds, such as *n*-alkanes and fatty acids, as carbon and energy sources. *Yarrowia lipolytica *can synthesize and accumulate large amounts of lipids, making it a promising host to produce various lipids and convert *n*-alkanes to useful compounds. For advanced use of *Y. lipolytica* in these applications, it is necessary to understand the metabolism of these hydrophobic compounds in this yeast and the underlying molecular mechanisms. In this review, current knowledge on the *n*-alkane metabolism and how this is regulated in *Y. lipolytica* is summarized. Furthermore, recent studies revealed that lipid transfer proteins are involved in the utilization of *n*-alkanes and the regulation of cell morphology in response to *n*-alkanes. This review discusses the roles of membrane lipids in these processes in *Y. lipolytica*.

## Introduction

*Yarrowia lipolytica* is a dimorphic yeast belonging to the phylum Ascomycota and is a particularly intriguing and important nonconventional yeast that can be utilized for basic science and biotechnological applications (Barth and Gaillardin 1996, 1997). *Yarrowia lipolytica* exhibits vigorous growth on various carbon sources, including glucose and glycerol, and has particularly outstanding abilities to assimilate hydrophobic substrates, such as *n*-alkane and triacylglycerol. *Yarrowia lipolytica* can synthesize and accumulate significant amounts of lipids in the cell, and therefore it is included in the so-called oleaginous yeasts. Although *Yarrowia lipolytica* is highly tolerant to various stresses, including those induced by salt, pH, and heavy metals (Mamaev and Zvyagilskaya 2021), it is generally unable to grow at temperatures > 32–35 °C. *Yarrowia lipolytica* is nonpathogenic to humans and has been certified as Generally Recognized As Safe (GRAS) by the U.S. Food and Drug Administration (Groenewald et al. 2014). Furthermore, a variety of molecular biological and genetic techniques are available in this yeast. Therefore, several attempts have been made in *Y. lipolytica* to construct systems to produce lipids and useful chemicals, including organic acids and polyunsaturated fatty acids (Lazar et al. 2018; Markham and Alper 2018; Miller and Alper 2019; Park and Ledesma-Amaro 2022). In addition, *Y. lipolytica* has an intrinsic ability to secrete large amounts of extracellular enzymes, including lipases and proteases, and has been considered as a potential host for secretory production of useful enzymes (Celińska and Nicaud 2019). In basic research, *Y. lipolytica* has been studied as a model organism to elucidate the molecular mechanisms underlying various cellular processes, such as peroxisome biosynthesis, dimorphism, and mitochondrial electron transport (Nicaud 2012).

A key characteristic feature of *Y. lipolytica* is the assimilation of *n*-alkanes. Various microorganisms, including bacteria, yeasts, and filamentous fungi, can utilize *n*-alkanes as carbon and energy sources. Approximately 180 yeast species, belonging to the Ascomycota and Basidiomycota phyla and including *Candida*, *Debaryomyces*, *Metschnikowia*, *Yarrowia*, and *Cryptococcus*, are reported to assimilate *n*-hexadecane, an *n*-alkane of 16 carbons (Kurtzman et al. 2011), and yeast *n*-alkane metabolism has been most deeply studied and elucidated in *Y. lipolytica*. Due to this ability, *Y. lipolytica* attracted attention for production of single cell protein (SCP) in the 1960s (Barth and Gaillardin 1996, 1997; Groenewald et al. 2014). In addition, *Y. lipolytica* has been studied for bioremediation of petroleum-contaminated soil and water (see review by Zinjarde et al. 2014), and for the biotransformation of *n*-alkanes to valuable compounds (Fickers et al. 2005). Particularly, conversion of *n*-alkanes to dicarboxylic acids, which are of industrial importance in the production of detergents, surfactants, lubricants, cosmetics, and plastic, using *Y. lipolytica* was attempted (Gatter et al. 2014; Smit et al. 2005).

This review focuses on the metabolism of *n*-alkanes and regulation of this in *Y. lipolytica*. In addition, the roles of membrane lipids and lipid transfer proteins in the *n*-alkane metabolism are discussed.

### Uptake of *n*-alkane

The mechanism whereby *Y. lipolytica* or other *n*-alkane-assimilating yeasts uptake *n*-alkanes, which are poorly soluble in water, remains poorly understood. Several yeasts are considered to secrete biosurfactant to solubilize hydrophobic substrates. For instance, *Starmerella bombicola* and *Candida apicola* produce sophorolipids (Van Bogaert et al. 2007). *Yarrowia lipolytica* was reported to secrete a 28-kDa emulsifier, named liposan, which is known to contain carbohydrate and protein (Cirigliano and Carman 1985), although the molecular structure and the involvement of liposan in the solubilization and uptake of *n*-alkanes remain unclear. Subsequently, various emulsifiers were reported from different strains (Zinjarde et al. 2014). *Yarrowia lipolytica* can adhere to *n*-alkane droplets probably through cell surface hydrophobic properties. The uptake of *n*-alkanes is possibly facilitated through this direct interaction of the cell with *n*-alkane droplets. Protrusions or slime-like outgrowths were reported to exist on the surfaces of the cells of *n*-alkane-assimilating yeasts such as *Candida tropicalis*, *Candida maltosa*, and *Y. lipolytica* cultured in medium containing *n*-alkane, and these protrusions or slime-like outgrowths were proposed to be involved in the attachment of the cells to *n*-alkanes (Kim et al. 2000; Mauersberger et al. 1996; Osumi et al. 1974).

A *Y. lipolytica* mutant containing an insertion mutation in *ABC1*, which encodes an ATP-binding cassette (ABC) transporter, exhibited defective growth on *n*-hexadecane, raising the possibility that *n*-hexadecane is incorporated by Abc1 (Thevenieau et al. 2007). However, the involvement of Abc1 in the uptake of *n*-hexadecane is yet to be investigated.

### Metabolic pathways of *n*-alkane

**Fig. 1:**
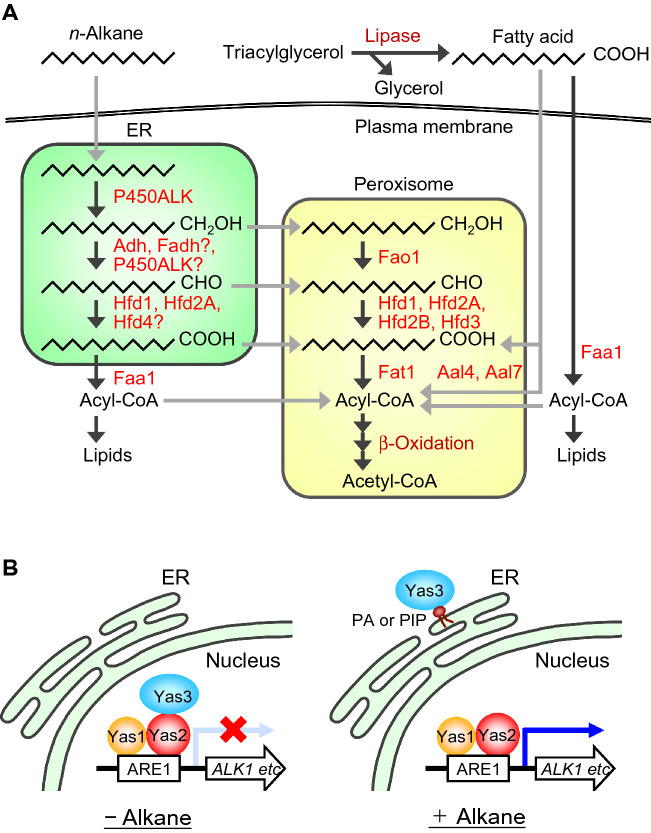
Metabolic pathway of *n*-alkane and triacylglycerol and transcription regulation of *n*-alkane metabolism in *Y. lipolytica*. **A** Metabolic pathway of *n*-alkane and triacylglycerol in *Y. lipolytica*. *n*-Alkanes are hydroxylated to fatty alcohols by cytochrome P450ALK belonging to the CYP52-family. Fatty alcohols are oxidized to fatty aldehydes by fatty alcohol dehydrogenase or P450ALK in the ER, or by fatty alcohol oxidase in the peroxisome. Roles of Fadh and P450ALK in the oxidation of fatty alcohol remain to be elucidated. Fatty aldehydes are oxidized to fatty acids by fatty aldehyde dehydrogenase in the ER or the peroxisome. Localization of Hfd4 remains unclear. Fatty acids are activated to acyl-CoAs by acyl-CoA synthetase, and are metabolized through β-oxidation pathway in the peroxisome or utilized for membrane or storage lipid synthesis in the ER. Triacylglycerol is hydrolyzed to glycerol and fatty acids by lipase. Fatty acids are incorporated and converted to acyl-CoAs by acyl-CoA synthetase, and metabolized or used for lipid synthesis. **B** Model of transcriptional regulation of *n*-alkane metabolism *Y. lipolytica*. Left: In the absence of *n*-alkanes, the transcription repressor Yas3p binds to the activator complex composed of Yas1p and Yas2p in the nucleus, and ARE1-dependent transcription is repressed. Right: In the presence of *n*-alkanes, Yas3p is sequestered to the ER via interaction with PA and/or PIP, and ARE1-dependent transcription is activated

Incorporated *n*-alkanes are transported to the endoplasmic reticulum (ER) and hydroxylated to fatty alcohols by cytochrome P450 (P450ALK), which belongs to the CYP52 family (Fig. 1A) (Fickers et al. 2005; Fukuda 2013; Fukuda and Ohta 2013, 2017a). *Yarrowia lipolytica* has 12 genes, *ALK1*–*ALK12*, in its genome that encode CYP52-family P450 enzymes (Hirakawa et al. 2009; Iida et al. 1998, 2000). Among these genes, the deletion mutant of *ALK1* showed significant growth defects when grown on media containing *n*-alkanes of 10 to 15 carbons (Iida et al. 1998; Takai et al. 2012). The *ALK2* deletion mutant did not exhibit growth defects on medium containing *n*-alkanes, but the double deletion mutant of *ALK1* and *ALK2* exhibited a severe growth defect on medium containing a 16-carbon *n*-alkane (Iida et al. 2000; Takai et al. 2012). These results indicate that Alk1 catalyzes the hydroxylation of *n*-alkanes of various chain lengths, playing a primary role in their assimilation, and that Alk2 has a hydroxylation activity for *n*-alkanes of longer chain lengths. A deletion mutant of all 12 *AKL* genes completely lost the ability to grow on medium containing *n*-alkanes, demonstrating the critical roles of P450ALKs in the hydroxylation of *n*-alkanes (Takai et al. 2012). A subset of Alk proteins can also hydrolyze the ω-terminus of dodecanoic acid. The CYP52-family P450s of *Y. lipolytica* can be classified into four groups: P450s with significant *n*-alkane-hydroxylating activities: Alk1, Alk2, Alk9, and Alk10; P450s with significant hydroxylating activities for the ω-terminus of dodecanoic acid: Alk4, Alk5, and Alk7; P450s with significant hydroxylating activities for both *n*-alkanes and dodecanoic acid: Alk3 and Alk6; and P450s with faint or no oxidizing activity for these substrates: Alk8, Alk11, and Alk12 (Iwama et al. 2016).

Fatty alcohols are thought to be oxidized to fatty aldehydes by NAD^+^- or NADP^+^-dependent fatty alcohol dehydrogenase (FADH) in the ER or by H_2_O_2_-producing fatty alcohol oxidase (FAOD) in the peroxisome (Fig. 1A) (Fickers et al. 2005; Fukuda 2013; Fukuda and Ohta 2013, 2017a). Eight alcohol dehydrogenase genes, *ADH1*–*ADH7*, *FADH*, and a fatty alcohol oxidase gene, *FAO1* are present in the *Y. lipolytica* genome. These genes are involved in the oxidation of hydroxyl groups in ω-hydroxy fatty acids in *Y. lipolytica* (Gatter et al. 2014). A triple deletion mutant of *ADH1*, *ADH3*, and *FAO1* exhibited severe growth defects on medium containing 1-dodecanol or 1-tetradecanol as a carbon source, suggesting that Adh1, Adh3, and Fao1 are involved in the utilization of fatty alcohols taken up from the medium (Iwama et al. 2015). Adh1 and Adh3 were suggested to localize in the cytosol, but these proteins may localize transiently to the ER. Fao1 localizes in the peroxisome. The deletion mutant of *ADH1*–*ADH7*, *FADH*, and *FAO1* showed slight growth defects on *n*-decane and *n*-dodecane, but not on *n*-alkanes of a longer chain length, indicating that other enzyme(s) is/are involved in the oxidation of fatty alcohols produced in the metabolism of *n*-alkanes.

Fatty aldehydes are oxidized to fatty acids by fatty aldehyde dehydrogenase (FALDH) in the ER or the peroxisome (Fig. 1A). *Yarrowia lipolytica* contains four genes, *HFD1*–*HFD4*, encoding FALDH, and a quadruplex deletion mutant of these genes showed severe growth defects on *n*-alkanes, although triple deletion mutants of any combination of these genes could grow on *n*-alkanes (Iwama et al. 2014). Among these gene products, Hfd1 localized to the ER and the peroxisome and Hfd3 localized to the peroxisome. Two transcription variants, *HFD2A* and *HFD2B*, were generated from *HFD2*, and the product of *HFD2A* localizes to the ER and the peroxisome, while that of *HFD2B* localizes to the peroxisome. The difference in the physiological roles of the ER- and the peroxisome-localized Hfd proteins and the significance of the alternative splicing of *HFD2* remain to be elucidated. Interestingly, whereas the triple deletion mutant that expresses *HFD1* or *HFD3* alone exhibited filamentous growth on *n*-hexadecane as the wild-type strain, the triple deletion mutant that expresses *HFD2* or *HFD4* alone had defective filamentous growth on medium containing *n*-hexadecane. This raises the possibility that Hfd proteins are involved in the transition of cell morphology in *Y. lipolytica*. In *Saccharomyces cerevisiae*, Hfd1, an orthologous protein of *Y. lipolytica* Hfd proteins, catalyzes the oxidation of hexadecenal, which is produced in the metabolism of sphingosine 1-phosphate, to hexadecenoic acid (Nakahara et al. 2012). Sphingosine 1-phosphate is an intermediate in the sphingolipid metabolic pathway and has signaling functions (Montefusco et al. 2014). Therefore, Hfd proteins may be involved in the regulation of cell morphology through the metabolism of sphingolipids.

Fatty acids are activated to acyl-CoAs by acyl-CoA synthetase (ACS) using coenzyme A and are used for membrane lipid synthesis or metabolized through β-oxidation in the peroxisome. *Y. lipolytica* has five ACS genes, *FAA1*, and *FAT1*–*FAT4*, and 10 ACS-like enzyme-coding genes, *AAL1*–*AAL10* (Dulermo et al. 2014, 2016; Tenagy et al. 2021, 2015; Wang et al. 2011). A double deletion mutant of *FAT1* and *FAA1* exhibited severely defective growth on 10–18 carbon *n*-alkanes, suggesting that Faa1 and Fat1 play critical roles in the activation of fatty acids produced during metabolism of *n*-alkanes (Tenagy et al. 2015). Faa1 localizes in the cytosol and to membranes, whereas Fat1 localizes in the peroxisome. Faa1 is involved in the activation of fatty acids that are produced in the metabolism of *n*-alkanes for membrane lipid synthesis.

Triacylglycerol is hydrolyzed to glycerol and fatty acids by lipases (Fig. 1A). *Yarrowia lipolytica* has 16 lipase genes, *LIP2*, *LIP4*, *LIP5*, and *LIP7*–*LIP19*, and 4 esterase genes, *LIP1*, *LIP3*, *LIP6*, and *LIP20*, in its genome (Fickers et al. 2013). Glycerol is a preferable carbon source for *Y. lipolytica*. Fatty acids are incorporated via unknown mechanism and utilized for lipid synthesis or metabolized as carbon and energy sources. Faa1 is involved in the activation of fatty acids incorporated from the culture medium for membrane lipid synthesis. ACS-like proteins, Aal1–Aal10, show sequence similarities not only to ACS but also to plant or bacterial 4-coumarate-CoA ligases and insect luciferases. All Aal proteins have peroxisomal targeting signal 1 (PTS1)-like sequences at their C-termini, and were suggested to have ACS activities (Dulermo et al. 2016). *AAL4* and *AAL7* exhibited the highest expression levels in the ACS-like genes when *Y. lipolytica* was cultured in medium containing oleic acid as a carbon source, and Aal4 and Aal7 were shown to be involved, together with Faa1 and Fat1, in the activation of exogenous fatty acids for utilization as carbon sources (Tenagy et al. 2021).

CYP52-family P450 genes have been identified in other yeasts that can assimilate *n*-alkanes, including *C. tropicalis* (Sanglard et al. 1987; Seghezzi et al. 1992, 1991), *C. maltosa* (Ohkuma et al. 1995, 1991), *Candida albicans* (Kim et al. 2007; Panwar et al. 2001), *Debaryomyces hansenii* (Yadav and Loper 1999), and *Starmerella bombicola* (Van Bogaert et al. 2009). In *C. maltosa*, the CYP52-family P450s are encoded by *ALK1*–*ALK8*, and the quadruple deletion mutant of *ALK1*, *ALK2*, *ALK3*, and *ALK5* showed defects in the utilization of *n*-alkanes, suggesting the involvement of these genes in the metabolism of *n*-alkanes (Ohkuma et al. 1998). Genes involved in the subsequent oxidation processes of *n*-alkane metabolism in other yeasts remain to be determined, but CYP52A3, a CYP52-family P450 of *C. maltosa*, was suggested to have the activity to catalyze the oxidation of fatty alcohols and fatty aldehydes (Scheller et al. 1998). Therefore, P450ALKs may catalyze the oxidation of fatty alcohols produced during *n*-alkane metabolism in *Y. lipolytica* (Fig. 1A).

### Transcriptional regulation of the genes involved in *n*-alkane metabolism

In *Y. lipolytica*, transcription of the genes involved in *n*-alkane metabolism, including *ALK1*, *ALK2*, *ALK6*, *ADH1*, *ADH3*, *FAO1*, *HFD1*–*HFD3*, *FAA1*, and *FAT1*, is activated by the presence of *n*-alkane (Fukuda and Ohta 2017b; Hirakawa et al. 2009; Iida et al. 1998, 2000; Iwama et al. 2014, 2015; Tenagy et al. 2015; Tezaki et al. 2017). Transcription of *ALK1* is upregulated by a complex of basic helix-loop-helix transcription factors, Yas1 and Yas2, in response to *n*-alkane, and expression is downregulated by the Opi1-family transcription repressor Yas3 (Fig. 1B) (Endoh-Yamagami et al. 2007; Fukuda 2013; Fukuda and Ohta 2013, 2017b; Hirakawa et al. 2009; Kobayashi et al. 2008; Yamagami et al. 2004). Yas1 and Yas2 constitutively localize in the nucleus and bind to the Alkane-Responsive Element 1 (ARE1) in the *ALK1* promoter. In the absence of *n*-alkane, Yas3 is targeted to the nucleus and binds to Yas2, resulting in the transcriptional repression of *ALK1*. In the presence of *n*-alkane, Yas3 is retained to the ER and the transcription of *ALK1* is activated. Although the mechanism whereby Yas3 is retained to the ER is unclear, Yas3 can bind to phosphatidic acid (PA) and phosphoinositides in vitro (Kobayashi et al. 2013, 2015) and could be retained to the ER through binding to PA or phosphoinositide (Fukuda 2013; Fukuda and Ohta 2017b).

*Yarrowia lipolytica* prefers glycerol to glucose as a carbon and energy source. In most organisms, the transcription of genes involved in using alternative carbon sources is repressed in the presence of glucose, which is often the most preferred carbon source. In contrast, the transcription of a subset of genes involved in *n*-alkane metabolism, including *ALK1*, is repressed by glycerol but not by glucose in *Y. lipolytica* (Mori et al. 2013). The molecular mechanisms underlying this peculiar transcription repression by glycerol are still awaiting discovery.

### Role of Sec14 family proteins in *n*-alkane utilization

The biological membranes of eukaryotic cells are composed of various lipid species, including phospholipids, sterols, and sphingolipids. These membrane lipids are synthesized only in specific organelles and are then present in the plasma membrane and most of the organelle membranes in specific proportions for each membrane. To maintain the lipid composition of the plasma membrane and organelle membranes, the transport of lipid molecules between membranes must be strictly executed. Intermembrane lipid transport is performed by vesicular and nonvesicular mechanisms. Nonvesicular lipid transport has been proposed to be mediated by lipid transfer proteins (LTPs) that extract a lipid molecule from one membrane and transfer and insert it to another membrane, and/or through membrane contact sites (MCSs), close appositions between two membranes (Egea 2021; Hanada 2018; Kors et al. 2022; Reinisch and Prinz 2021; Wong et al. 2019). Major eukaryotic LTPs include the Sec14-family proteins, oxysterol-binding protein (OSBP)-related proteins (ORPs), StARkin superfamily proteins, chorein_N motif proteins, and TULIP superfamily proteins. Here the roles of the Sec14-family proteins and ORPs in the *n*-alkane metabolism of *Y. lipolytica* are discussed.

**Fig. 2:**
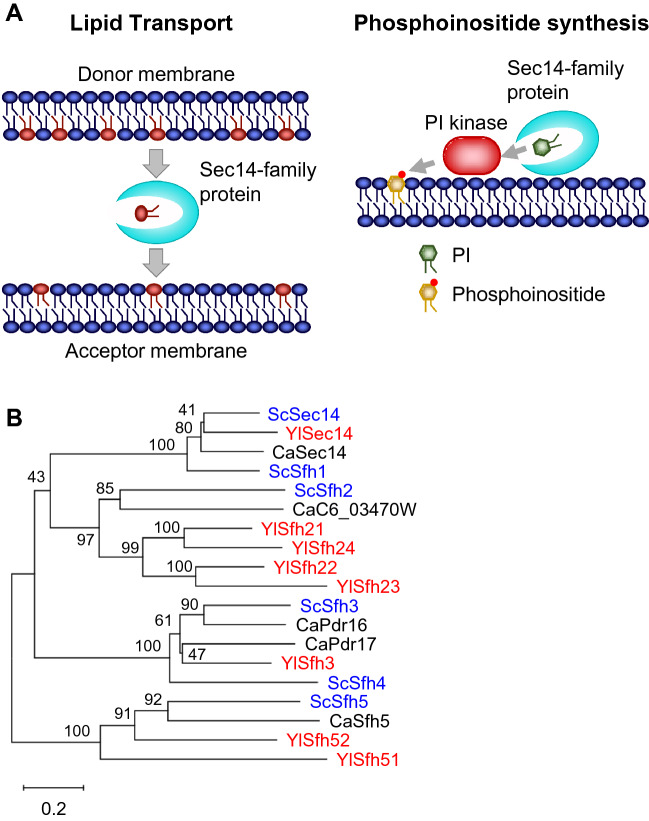
Model of the function of Sec14-family protein and phylogenetic tree of Sec14-family proteins of *S. cerevisiae*, *C. albicans*, and *Y. lipolytica*. **A** Model of the function of Sec14-family protein. *Left panel*, Sec14-family protein transports lipids between membranes. *Right panel*, Sec14-family protein presents PI to PI kinase to produce phosphoinositide. See text in detail. **B** The phylogenetic tree of Sec14-family proteins of *S. cerevisiae* (Sc), *C. albicans* (Ca), and *Y. lipolytica* (Yl) was constructed using MEGAX. The scale bar denotes 0.2 substitutions per site. The bootstrap frequencies are indicated. The accession numbers of sequences from UniProtKB are as follows: Sec14 (P24280), Sfh1 (P33324), Sfh2 (Q06705), Sfh3 (P53860), Sfh4 (P53844), and Sfh5 (P47008) of *S. cerevisiae*, Sec14 (P46250), C6_03470W (A0A1D8PQ60), Pdr16 (A0A1D8PD50), Pdr17 (A0A1D8PGP3), and Sfh5 (A0A8H6BRU9) of *C. albicans*, and Sec14 (P45816), Sfh21 (Q6CBL6), Sfh22 (Q6C4V3), Sfh23 (Q6CCY6), Sfh24 (Q6C4B2), Sfh3 (Q6CHI5), Sfh51 (Q6C1G2), and Sfh52 (Q6C9R9) of *Y. lipolytica*

In *S. cerevisiae*, *SEC14* encodes a protein that can transport phosphatidylinositol (PI) and phosphatidylcholine (PC) between membranes in vitro (Bankaitis et al. 1990). There are five additional Sec14-family protein genes, *SFH1*–*SFH5*, in the genome of *S. cerevisiae*. Although *SEC14* is essential for growth and secretion, *SFH1*–*SFH5* are nonessential. Sfh2–Sfh5 exhibited transfer protein activities in vitro for PI but not for PC (Li et al. 2000). The physiological roles of Sec14-family proteins remain under debate, and those proteins are proposed to be involved in intracellular signaling through promotion of phosphoinositide synthesis by presenting PI to PI kinases (Fig. 2A) (Grabon et al. 2019). In contrast, the Sec14-family proteins may function as LTPs that mediate phospholipid transport between membranes (Fig. 2A) (Lipp et al. 2020). In accordance with the latter model, we previously showed that Sfh1 of *S. cerevisiae* can transport phosphatidylserine (PS) between membranes in vitro (Mizuike et al. 2019).

Sec14-family proteins are also conserved in other yeasts (Holič et al. 2021). Eight genes encoding Sec14-family proteins are present in the genome of *Y. lipolytica* (Fig. 2) (Watanabe et al. 2022). In *Y. lipolytica*, *SEC14* is nonessential for growth or secretion (Lopez et al. 1994) but this is essential for growth in *C. albicans*, similar to the requirement in *S. cerevisiae* (Monteoliva et al. 1996). The deletion mutant of *SEC14* of *Y. lipolytica* exhibited a defect in the filamentous growth in rich medium, suggesting a role of *SEC14* in cell morphology transition in *Y. lipolytica* (Lopez et al. 1994). Another intriguing feature of the *SEC14*-family genes of *Y. lipolytica* is that *Y. lipolytica* contains four *SFH2* orthologs, *SFH21*–*SFH24*, and two *SFH5* orthologs, *SFH51* and *SFH52*, in contrast to *S. cerevisiae* and *C. albicans*, both of which have only one ortholog each of *SFH2* and *SFH5*. Interestingly, the transcription of *SFH21*–*SFH23* and *SFH51* is upregulated in response to *n*-alkanes in *Y. lipolytica* (Watanabe et al. 2022). Filamentous growth is highly induced when *Y. lipolytica* is cultured on solid medium containing *n*-alkanes as carbon sources. The deletion mutant of *SFH21* exhibited severe defects during growth on *n*-alkanes and filamentous growth in response to *n*-alkanes, and additional deletion of *SFH22* and *SFH23* exacerbated those defects (Watanabe et al. 2022). These results suggest that the *SFH2* orthologs in *Y. lipolytica* are involved in the utilization of *n*-alkanes and the transition of cell morphology in response to *n*-alkanes. The orthologous proteins of Sfh2 may function as LTPs that mediate phospholipid transport between membranes in the cells, and the deletion of *SFH2* orthologs could prevent maintenance of the lipid composition of the plasma membrane and/or organelle membranes appropriate for *n*-alkane metabolism in *Y. lipolytica*. Sfh2 of *S. cerevisiae* has an activity to transport squalene, a precursor of sterol synthesis, in vitro (Tripathi et al. 2019), and it is therefore possible that the orthologous proteins of Sfh2 are involved in maintaining sterol levels in the membrane. During *n*-alkane metabolism, hydrophobic substrates need to be transported between membranes, and *n*-alkane is transported from the plasma membrane to the ER while fatty alcohol, fatty aldehyde, and/or fatty acid are/is transported from the ER to the peroxisome. However, the mechanisms for intermembrane transport of these hydrophobic substrates remain to be elucidated. The orthologous proteins of Sfh2 could transport hydrophobic substrates between the plasma membrane and the ER or between the ER and the peroxisome in *Y. lipolytica*. Alternatively, the orthologous proteins of Sfh2 may present PI to PI kinases for phosphoinositide synthesis, and phosphoinositides could be involved in the metabolism of *n*-alkane or its regulation in *Y. lipolytica*. As mentioned above, the transcriptional repressor Yas3 bound to PA and phosphoinositides in vitro (Kobayashi et al. 2013, 2015). The orthologous proteins of Sfh2 could regulate *n*-alkane metabolism and morphogenesis in response to *n*-alkane through promotion of phosphoinositide synthesis. However, it does not appear to be feasible that the orthologous proteins of Sfh2 regulate the transcription of *ALK* by promoting phosphoinositide synthesis because the deletion mutant of *SFH21* did not show defects in the transcription activation of *ALK1* by *n*-alkanes (Watanabe et al. 2022).

### Role of oxysterol-binding protein homologs in *n*-alkane utilization

**Fig. 3:**
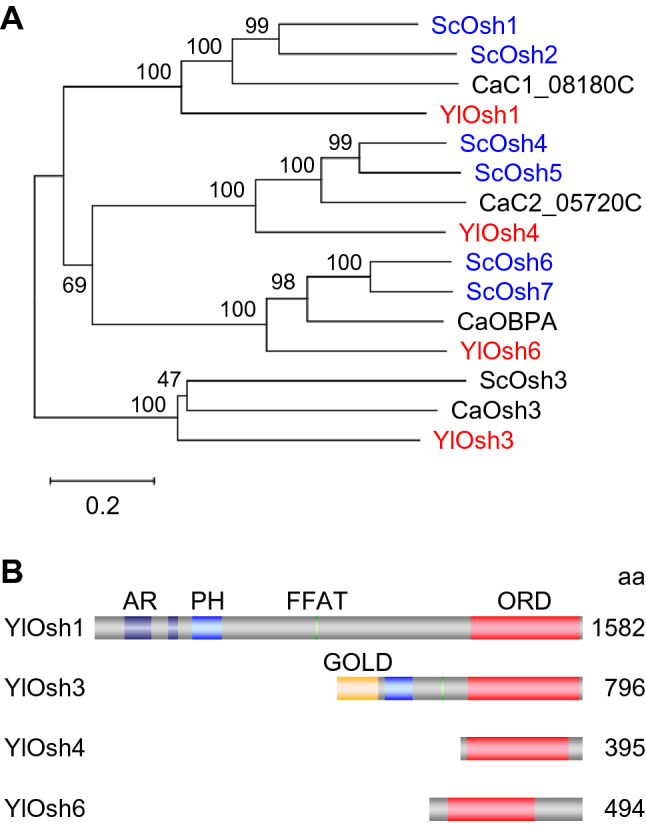
Phylogenetic tree of ORPs of *S. cerevisiae*, *C. albicans*, and *Y. lipolytica* and structures of ORPs of *Y. lipolytica*. **A** The phylogenetic tree of ORPs of *S. cerevisiae* (Sc), *C. albicans* (Ca), and *Y. lipolytica* (Yl) was constructed using MEGAX. The scale bar denotes 0.2 substitutions per site. The bootstrap frequencies are indicated. The accession numbers of sequences from UniProtKB are as follows: Osh1 (P35845), Osh2 (Q12451), Osh3 (P38713), Osh4 (P35844), Osh5 (P35843), Osh6 (Q02201), and Osh7 (P38755) of *S. cerevisiae*, C1_08180C (A0A1D8PE79), Osh3 (Q59TM0), C2_05720C (A0A1D8PHG1), and OBPA (A0A8H6F3A4) of *C. albicans*, and Osh1p (Q6C1E8), Osh3p (Q6C4Y2), Osh4p (Q6CC78), and Osh6p (Q6CI24) of *Y. lipolytica*. **B** Structures of ORPs of *Y. lipolytica*. Oxysterol-binding protein domain, Pleckstrin homology domain, FFAT motif, Ankyrin repeat, and GOLD domain are shown

ORPs are widely conserved LTPs in eukaryotes (Arora et al. 2022), seven genes for OSBP homologs, *OSH1*–*OSH7*, are present in the *S. cerevisiae* genome (Fig. 3). Although OSBP was originally identified as a protein that binds to oxysterol, several ORPs were reported to bind cholesterol or ergosterol as well. ORPs contain an OSBP-related ligand-binding domain (ORD) that is involved in lipid-binding. In *S. cerevisiae*, none of *OSH* genes are essential for growth, although the deletion of seven *OSH* genes resulted in lethality, indicating that Osh proteins share an overlapping essential function (Beh et al. 2001) that is currently undetermined. Among the Osh proteins, Osh4 could transport sterol between membranes in vitro (Raychaudhuri et al. 2006), and subsequently it was suggested that all Osh proteins share this ability (Tian et al. 2015, 2018). Conversely, Osh6 and Osh7 were shown to transport PS from the ER to the plasma membrane (Maeda et al. 2013). Furthermore, Osh proteins were reported to function as lipid sensors or regulators in various processes, including exocytosis (Alfaro et al. 2011), plasma membrane sterol organization (Georgiev et al. 2011), phosphoinositide metabolism (Stefan et al. 2011), TORC1 signaling and nitrogen sensing (Mousley et al. 2012), and phospholipid synthesis (Tavassoli et al. 2013),. Therefore, the molecular functions of ORPs remain to be established.

ORPs are conserved in fungi (Qiu and Zeng 2019), and *Y. lipolytica* has four ORP genes, *OSH1*, *OSH3*, *OSH4*, and *OSH6* (Fig. 3A). Osh1 and Osh3 of *Y. lipolytica* have N-terminal extensions containing various domains or motifs that interact with lipids or other proteins in addition to the ORD, similarly to Osh1–Osh3 of *S. cerevisiae*, whereas Osh4 and Osh6 of *Y. lipolytica* mainly consist of the ORD as do Osh4–Osh7 of *S. cerevisiae* (Fig. 3B). Among the four *OSH* genes, the deletion mutant of *OSH3* or *OSH6* exhibited growth defects on *n*-alkanes. In the *OSH6* deletion mutant, the *ALK1* transcription was upregulated in response to *n*-alkane, but functional P450 was not produced (Iwama et al. 2018). It is unclear whether Osh6 of *Y. lipolytica* transports sterol, PS, or other lipids between membranes, although it is possible that the lipid composition of the ER membrane, where the P450ALKs localize and function, is altered in the absence of *OSH6*, resulting in the defects in the folding or activity of the P450ALKs. In contrast, because functional P450 was produced in the deletion mutant of *OSH3* in response to *n*-alkane (unpublished results), Osh3 could help maintain correct lipid compositions of other membranes, e.g., the plasma membrane. Alternatively, Osh3 could mediate intracellular transport of *n*-alkane or its metabolites between membranes. The deletion mutant of *OSH3* of the *S. cerevisiae* Σ1278b strain was reported to show enhanced filamentous growth, while deletion of *OSH3* caused a defect in the filamentous growth in *C. albicans* (Hur et al. 2006), raising the possibility that *OSH3* is involved in the transition of cell morphology in *Y. lipolytica*.

## Conclusions and future perspectives

The metabolic pathway of *n*-alkanes and most of the enzymes that catalyze the reactions in *Y. lipolytica n*-alkane metabolism have been elucidated. In contrast, how *n*-alkane is transported from the plasma membrane to the ER and how *n*-alkane metabolites migrate from the ER to the peroxisome are open questions. *n*-Alkane and its metabolites are highly hydrophobic, and it is less probable that they move between membranes by free diffusion in the cytosol. Thus, transport by LTPs or via MCSs is a candidate mechanism for transporting *n*-alkane and its metabolites between membranes, as well as membrane lipids. Currently it is unclear whether Sec14-family proteins and ORPs are involved in the intermembrane transport of *n*-alkane or its metabolites. In addition to Sec14-family proteins and ORPs, other LTPs exist in eukaryotes and it would be interesting to examine the growth of the deletion mutants of these LTP genes on *n*-alkanes. Furthermore, involvement of MCSs in the *n*-alkane utilization is intriguing. Studies on the transport of *n*-alkane and its metabolites in *Y. lipolytica* are expected to provide a useful model to understand the intracellular transport of hydrophobic metabolites in eukaryotes.

The roles of membrane lipids in the response to *n*-alkane and its metabolism in *Y. lipolytica* are currently underexplored. The lipid composition of ER and the peroxisomes may affect the folding or the activity of the enzymes involved in the *n*-alkane metabolism. In addition, the lipid composition of the plasma membrane, including signaling lipids such as phosphoinositide, could be important in regulating cell morphology and in responding to the stresses caused by these hydrophobic substrates. To clarify the roles of membrane lipids in the response to *n*-alkane and its metabolism, it is important to construct a system to analyze the lipidome of the plasma membrane and the organellar membranes and their local alteration in response to carbon sources. In the *n*-alkane-assimilating yeasts *C. tropicalis* and *C. maltosa*, *n*-alkane was shown to induce the proliferation of the ER and peroxisome (Mauersberger et al. 1987; Osumi et al. 1974). Although the molecular mechanism of the peroxisome biogenesis has been well-established, that of the ER proliferation remains poorly understood. Quantitative analysis of the organellar membranes is also important to understand the response of *Y. lipolytica* to *n*-alkanes.

Several proteins, including transcription factors, Mhy1, Hoy1, Tec1, Rim101, Fts1, Fts2, Nrg1, Tup1, and Ssn6, and regulators, Ras2, Rac1, and Bmh1, are shown to be associated with the transition of cell morphology in *Y. lipolytica* (Chen et al. 2022; Hurtado et al. 2000; Hurtado and Rachubinski 1999, 2002a, b; Mao et al. 2023; Richard et al. 2001; Shu et al. 2021; Torres-Guzmán and Domínguez 1997; Zhao et al. 2013). The relationships between these proteins and the LTPs are issues to be elucidated in the future.

As stated in the introduction, *Y. lipolytica* has a variety of advantageous features for industrial use. Elucidation of the metabolism of hydrophobic compounds and its regulation in *Y. lipolytica* will contribute to enabling advanced use of this yeast as a host for lipid production and bioconversion of hydrophobic substrates to various chemicals.
